# The emerging roles of lysine-specific demethylase 4A in cancer: Implications in tumorigenesis and therapeutic opportunities

**DOI:** 10.1016/j.gendis.2022.12.020

**Published:** 2023-03-23

**Authors:** Guanjun Yang, Changyun Li, Fan Tao, Yanjun Liu, Minghui Zhu, Yu Du, Chenjie Fei, Qiusheng She, Jiong Chen

**Affiliations:** a State Key Laboratory for Managing Biotic and Chemical Threats to the Quality and Safety of Agro-products, Ningbo University, Ningbo, Zhejiang 315211, China; b School of Life Science and Engineering, Henan University of Urban Construction, Pingdingshan, Henan 467044, China; c Laboratory of Biochemistry and Molecular Biology, School of Marine Sciences, Ningbo University, Ningbo, Zhejiang 315211, China; d Key Laboratory of Aquacultural Biotechnology Ministry of Education, Ningbo University, Ningbo, Zhejiang 315211, China

**Keywords:** Cancer therapy, Drug resistance, Histone demethylation, JmjC domain, KDM4A

## Abstract

Lysine-specific demethylase 4 A (KDM4A, also named JMJD2A, KIA0677, or JHDM3A) is a demethylase that can remove methyl groups from histones H3K9me2/3, H3K36me2/3, and H1.4K26me2/me3. Accumulating evidence suggests that KDM4A is not only involved in body homeostasis (such as cell proliferation, migration and differentiation, and tissue development) but also associated with multiple human diseases, especially cancers. Recently, an increasing number of studies have shown that pharmacological inhibition of KDM4A significantly attenuates tumor progression *in vitro* and *in vivo* in a range of solid tumors and acute myeloid leukemia. Although there are several reviews on the roles of the KDM4 subfamily in cancer development and therapy, all of them only briefly introduce the roles of KDM4A in cancer without systematically summarizing the specific mechanisms of KDM4A in various physiological and pathological processes, especially in tumorigenesis, which greatly limits advances in the understanding of the roles of KDM4A in a variety of cancers, discovering targeted selective KDM4A inhibitors, and exploring the adaptive profiles of KDM4A antagonists. Herein, we present the structure and functions of KDM4A, simply outline the functions of KDM4A in homeostasis and non-cancer diseases, summarize the role of KDM4A and its distinct target genes in the development of a variety of cancers, systematically classify KDM4A inhibitors, summarize the difficulties encountered in the research of KDM4A and the discovery of related drugs, and provide the corresponding solutions, which would contribute to understanding the recent research trends on KDM4A and advancing the progression of KDM4A as a drug target in cancer therapy.

## Introduction

Epigenetics refers to heritable changes in gene expression and function without affecting DNA sequence.^1^^,^^2^ Histone demethylation is one of the key epigenetic modifications mediating chromatin remodeling and thus regulating gene transcription.^3^, ^4^, ^5^, ^6^ Lysine-specific demethylase 4 A (KDM4A), also named Jumonji domain 2 A (JMJD2A), KIA0677, or JmjC domain-containing histone demethylation protein 3 A (JHDM3A), is an α-ketoglutaric acid (2-OG)- and Fe(II)-dependent histone-specific demethylase.^5^ It removes di- and tri-methyl groups from H3K9me2/3, H3K36me2/3, and H1.4K26me2/me3 and thus regulates transcriptional repression or activation *in cellulo*.^7^, ^8^, ^9^, ^10^, ^11^ Mechanistically, KDM4A catalyzes the oxidative decarboxylation of 2-OG by consuming O_2_ to generate a reactive iron (IV)-oxo intermediate, carbon dioxide, and succinate. Subsequently, the hemiaminal of the methylated lysine residue fragments liberates both formaldehyde and an unmethylated lysine residue.^7^^,^^9^ KDM4A is associated with many physiological processes, such as the differentiation of adipogenic, osteogenic, neural stem, and embryonic stem cells,^12^, ^13^, ^14^ the development of germ cells and embryos,^15^, ^16^, ^17^ the regeneration of skeletal muscle,^18^ and the activation of B cells.^19^ It is also involved in many non-cancer diseases, including diabetes,^20^ cardiac hypertrophy,^21^ atherosclerosis,^22^ viral infection,^23^, ^24^, ^25^, ^26^ Alzheimer's disease,^27^ colitis,^28^ and systemic lupus erythematosus (SLE).^19^ In addition, KDM4A is aberrantly expressed in many cancers and mediates their progression.^8^^,^^10^ Therefore, KDM4A is a potential therapeutic and diagnostic target for various cancers.^7^^,^^9^

Herein, the structure and functions of KDM4A in homeostasis and noncancer diseases are first introduced. Then, the role of KDM4A in the tumorigenesis of various cancers is summarized in detail. In addition, the classification of KDM4A inhibitors and their therapeutic mechanisms for cancer, as well as the current challenges and potential opportunities of KDM4A for anticancer therapy, are discussed.

## The structure and function of KDM4A

### The overview structure of KDM4A

**Figure 1:**
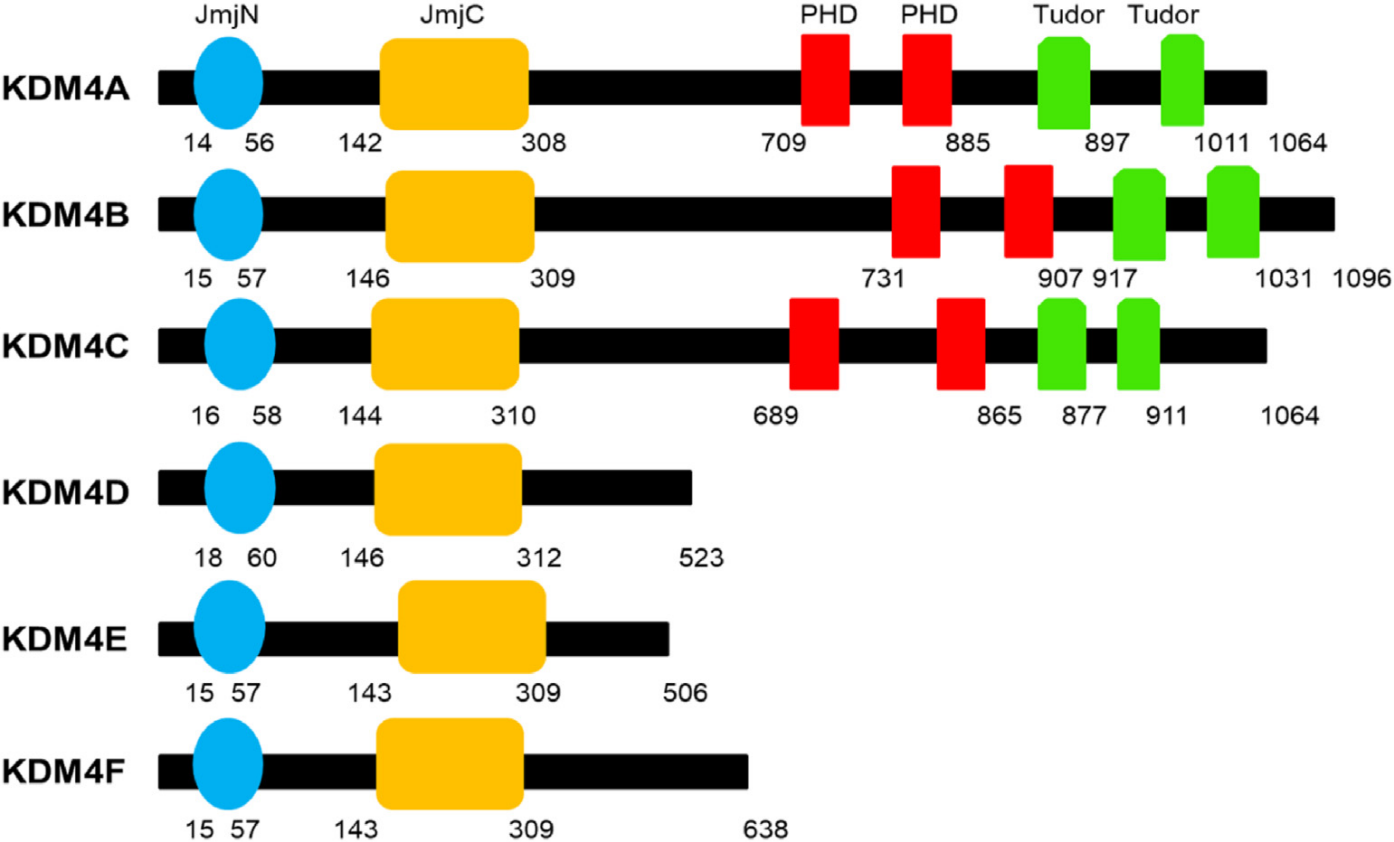
Schematic of the domains of KDM4s.

KDM4A belongs to the KDM4 subfamily, which consists of 6 members: KDM4A, KDM4B, KDM4C, KDM4D, KDM4E, and KDM4F.^8^ All KDM4s have their evolutionarily conserved domains Jumonji N (JmjN) and JmjC in common and share H3K9/K36me2/3-selective demethylase activities.^9^ KDM4A-Cs share two plant homeodomain (PHD) domains and two Tudor domains, while KDM4D-Es lack the two kinds of domains.^9^ The JmjN and JmjC domains form a catalytic histone demethylase domain, which removes the di- and tri-methylated groups from histone H3 lysine 9 (H3K9me2/me3) (Fig. 1). Although all KDM4s function as demethylases with the same substrates, their activities are dramatically distinct from each other, with several KDM4s even exhibiting weak demethylase activity against H1.4K26me3, H3K36me3, and H3K27me2/3 *in vitro*.^8^ KDM4A-Cs are more efficient at demethylating H3K9me3/H3K36me3 than H3K9me2/H3K36me2, whereas KDM4D is more efficient at demethylating H3K9me2 than H3K9me3. Through these specific activities, KDM4 stimulates or represses the expression of specific target genes. Heterochromatin regions that are condensed and transcriptionally silent are generally enriched in H3K9me3, and loss or reduction of H3K9me3 causes this facultative heterochromatin to decondense and become transcriptionally permissive. Therefore, overexpression of KDM4 can lead to reduced H3K9me3 levels and activate H3K9me3-mediated repressive genes.

### The function of KDM4A

Like other KDMs, KDM4A is also broadly expressed in multiple normal tissues and is involved in diverse biological processes in demethylase-dependent or demethylase-independent modes in both homeostasis and diseases.^18^^,^^27^^,^^29^, ^30^, ^31^, ^32^, ^33^, ^34^, ^35^

### KDM4A in normal physiology

As an epigenetic eraser, KDM4A plays pivotal roles in many physiological processes and mediates cell proliferation, differentiation, stemness, and development in a demethylase-dependent or demethylase-independent manner (Table 1). In muscle development and regeneration, KDM4A promotes myoblast proliferation and differentiation and myotube formation in myogenesis by erasing the histone repressive methylation mark H3K9me3 and thus transcriptionally activating *MyoD*, *MyoG*, and *Myf5*.^18^ Interestingly, an isoform KDM4A lacking the N-terminal demethylase domain can also promote muscle cell differentiation by increasing *MyoG* levels.^36^ In addition, KDM4A is associated with the proliferation and differentiation of stem cells.^14^^,^^37^, ^38^, ^39^ The H3K9me3-demethyalase activity of KDM4A is crucial for the self-renewal and early development of embryonic stem cells (ESCs).^15^^,^^40^ It promotes capillary tube formation and vasculogenesis by modulating the differentiation of ESCs into endothelial cells by occupying the *Flk 1* promoter and activating its transcription.^14^ The overexpression of KDM4A significantly improves the efficacy of human somatic cell nuclear transfer (SCNT) and promotes the derivation of pluripotent stem cells via H3K9me3-demethylase activity.^31^^,^^39^ In pig cloning by SCNT, KDM4A improves the development of preimplantation stage embryos by inducing *XIST* derepression.^38^ Moreover, KDM4A was also found to control the sequential activation of neural, neural crest, and sensory progenitor determinants by transcriptionally activating *PRDM1*.^37^ It mediates the differentiation of neural crest and neural stem cells (NSCs) by fine-regulating downstream genes in a demethylase-dependent manner.^13^^,^^41^ KDM4A promotes neural crest development by activating the KDM4A/H3K9me3/Sox 10 axis and inactivating the KDM4A/H3K36me3/Snail 2 axis.^41^ It has also been found to induce NSC differentiation into neurons by increasing *BDNF* levels by inactivating the repressive marker H3K9me3 and suppressing NSC differentiation into astroglia by reducing *GFAP* levels.^13^ Moreover, KDM4A has also been found to modulate adipogenic and osteogenic differentiation by up-regulating secreted frizzled-related protein 4 (Sfrp4) and CCAAT/enhancer-binding protein α (C/EBPα). The overexpression of Sfrp4 and C/EBPα contributes to suppressing osteogenic differentiation and facilitating adipogenic differentiation.^12^ Further study showed that KDM4A inhibits osteogenic differentiation by reducing the levels of *Runx 2*, *Osterix*, and *OCN*.^42^

**Table 1:** The roles of KDM4A in normal physiology.

Substrates	Target genes	Functions	Reference
H3K9me3	*MyoD*, *MyoG*, *Myf5*	Promoting myogenesis	^18^
H3K9me3	*Flk 1*	Promoting capillary tube formation and vasculogenesis	^14^
H3K9me3	*XIST*	Improving the development of preimplantation stage embryos	^38^
H3K9me3	*PRDM1*	Controlling the sequential activation of neural, neural crest, and sensory progenitor determinants	^37^
H3K4me9, H3K36me3	*Sox 10*, *Snail 2*	Promoting neural crest development	^41^
H3K9me3	*BDNF*	Inducing NSCs differentiation into neurons	^13^
H3K9me3	*GFAP*	Suppressing NSC differentiation into astroglia	^13^
H3K9me3	*Sfrp4*, *C/EBPα*	Suppressing osteogenic differentiation and facilitating adipogenic differentiation	^12^
H3K9me3	*Runx 2*, *Osterix*, *OCN*	Inhibiting osteogenic differentiation	^42^

### KDM4A in noncancer pathological diseases

Apart from physiological functions, KDM4A is also involved in modulating several non-cancer diseases by regulating different downstream genes (Table 2), such as cardiac hypertrophy,^21^ atherosclerosis,^22^ microbial infection,^23^, ^24^, ^25^^,^^43^ SLE,^19^ ischemic stroke,^44^ vascular inflammation,^20^^,^^45^ liver fibrosis,^21^ and mood disorders.^46^ Four-and-a-half LIM domain 1 (FHL1) is a key component of the mechano-transducer machinery in the heart, and KDM4A can occupy the *FHL1* promoter in response to transverse aortic constriction and up-regulate FHL1 levels by removing methyl groups from H3K9me3 during cardiac hypertrophy.^21^ The proliferation and migration of vascular smooth muscle cells (VSMCs) are two crucial cell events in atherosclerosis, and KDM4A was found to promote these two processes by inhibiting cyclin D1 expression and increasing p21 expression in a demethylase-dependent manner.^22^ The switch between viral latency and lytic cycles is often accompanied by specific alterations in histone codes, and KDM4A mediates this process by mediating the reactivation and replication of these viruses by interacting with distinct ligand proteins. During infection by Kaposi's sarcoma-associated herpesvirus (KSHV), KDM4A is SUMOylated at Lys 471 by the SUMO-2/3-specific E3 ligase KSHV K-bZIP in a SUMO-interacting motif (SIM)-dependent manner, and this modification is indispensable for KDM4A-mediated stabilization of chromatin association and gene transactivation.^25^^,^^26^ Further study indicated that SUMOylated KDM4A is required for the survival, movement, and angiogenesis of lytic KSHV-infected primary effusion lymphoma cells by modulating IL-10 levels.^43^ In addition, KDM4A also regulates the replication of human T-cell lymphotropic virus 1 (HTLV-1) and human papillomavirus (HPV) via interaction with H3K36me3 and retinoblastoma protein (pRb), respectively.^24^^,^^47^ In SLE, depletion of KDM4A and KDM4C potentiates B-cell activation and proliferation in response to *T* follicular helper cell-derived signals by reducing the levels of the cell cycle inhibitors Cdkn2c and Cdkn3.^19^ KDM4A reduces functional recovery in ischemic stroke by activating NF-κB downstream genes and subsequent neuroinflammation.^44^ In vascular smooth muscle cells, KDM4A induces vascular inflammation by increasing the levels of *IL-6* and *MCP-*1 by enhancing the occupation of H3K9me3 on their promoters.^20^^,^^45^ KDM4A has also been found to modulate hepatic stellate cell activation and liver fibrosis by epigenetically regulating peroxisome proliferator-activated receptor γ (PPARγ).^21^ KDM4A promotes glial activation in neonatal mice and causes adult mood disorders through epigenetic regulation.^46^

**Table 2:** The roles of KDM4A in noncancer pathological diseases.

Substrates	Target genes	Functions	References
H3K9me3	*FHL1*	Promoting cardiac hypertrophy	^21^
H3K9me3	*Cyclin D1*	Promoting proliferation and migration of VSMCs	^22^
	*p21*		
H3K9me3	*IL-10*	Maintaining survival, movement, and angiogenesis of lytic KSHV-infected primary effusion lymphoma cells	^43^
H3K9me3	*Cdkn2c*, *Cdkn3*	Potentiating B-cell activation and proliferation	^19^
H3K9me3	*NF-κB downstream genes*	Reducing functional recovery in ischemic stroke	^44^
H3K9me3	*IL-6*, *MCP-1*	Inducing vascular inflammation	^20^,^45^
H3K9me3	*PPARγ*	Modulating hepatic stellate cells activation and liver fibrosis	^12^
H3K9me3	*Runx 2*, *Osterix*, *OCN*	Inhibiting osteogenic differentiation	^42^

### KDM4A in cancer

KDM4A is also aberrantly expressed in multiple cancers and contributes to tumorigenesis and drug resistance by transcriptionally modulating different effector proteins (Table 3).

**Table 3:** The roles of KDM4A in tumorigenesis.

Cancer type	Target genes/ligand proteins	Functions	References
Leukemia	*TPM2*, *SLC29A2*, *CD82*	Promoting promotes their self-renewal, proliferation, and survival during leukemogenesis, and sensitizing to chemical agents	49, 50, 51, 52, 53
Lung cancer	*CHD5*, *CXCL5*, *ADAM12*, *JAG1*	Promoting transformation, proliferation, invasion, metastasis, and drug resistance	62, 63, 64
Breast cancer	*c-Jun*, *cyclin D1*	Promoting progression of breast cancer, and associated with breast cancer grade, TNM stage, histological type, and disease-free survival	^104^
PCa	*PSA*, *YAP1*, *MMP1*, *PSMD10*, *MMP7*, *PDK1*, *PDK3*	Promoting the progression of prostate cancer	90, 91, 92^,^^94^, ^95^, ^96^, ^97^, ^98^, ^99^
GC	CCDC8	Promoting growth and invasion of GC and sensitizing GC cells to cisplatin, 5-FU, and docetaxel	^102^,^104^
HCC	*miR-372*, *Pim 1*, *p21*	Accelerating malignant progression of HCC	106, 107, 108
CRC	*RASSF1A*, 53BP1	Sensitizing chemotherapy-induced cell death and leading to genomic integrity	^110^,^112^,^113^
PDAC	*miR-137*, RFXAP	Promoting proliferation	^114^,^115^
Bladder cancer	*ADAM12*, *SLUG*	Promoting muscle invasion, extravesical extension, and lymph node metastasis	^62^,^118^
Cervical cancer	*miR-491-5p*, *HIF1α*, *TfR1*, *DMT1*	Promoting proliferation and inhibiting ferroptosis	^119^,^120^
Renal carcinoma	Ribosomal protein-coding genes	Sustaining tumor cell survival under amino acid deprivation	^121^
OS	*SLC7A11*	Promoting drug resistance and lung metastasis by reducing ferroptosis	^122^
HNSCC	*JUN and FOS*	Promoting invasion, and metastasis	^124^
EC	*c-MYC*, *AR*, *p27*	Promoting proliferation, invasion, and metastasis	^126^,^127^

### KDM4A in leukemia

Leukemia is characterized by the malignant proliferation of hematopoietic stem cells (HSCs) in the bone marrow, producing a large number of cancer cells that are stagnant at various stages of cell development.^48^ KDM4A is found in several subtypes of leukemia and mediates their self-renewal, proliferation, and survival during leukemogenesis.^35^^,^^49^, ^50^, ^51^, ^52^ In acute myeloid leukemia (AML), KDM4A is overexpressed and promotes the KDM4A-PAF1 signaling-mediated transcriptional program by removing methyl groups from H3K9me3 and H3K9me27, regulating 9 genes associated with poor prognosis of AML and thus maintaining AML self-renewal and survival, while KDM4A knockout can induce apoptosis of AML cells.^51^ In chronic lymphocytic leukemia (CLL), KDM4A was also overexpressed in CLL samples compared with control samples.^50^ In relapsed acute lymphoblastic leukemia (ALL), H3K36me3, a substrate of KDM4A, localizes components of the DNA damage response (DDR) pathway and induces resistance to DNA damage agents (doxorubicin, etoposide, 6-thioguanine, and cytarabine), while KDM4A inhibition could restore the H3K36me3 level and sensitize ALL cells to cytarabine.^53^

### KDM4A in lung cancer

Lung tumors are the leading type of malignant tumor that cause the death of cancer patients worldwide every year.^54^ KDM4A is aberrantly expressed in lung carcinoma and mediates tumorigenesis by modulating the transformation, proliferation, invasion, and metastasis of cancer cells.^55^, ^56^, ^57^, ^58^, ^59^, ^60^, ^61^, ^62^, ^63^ In the human lung cancer cell line A549, KDM5A collaborates with oncogenic K-Ras to promote cellular transformation by down-regulating tumor suppressor chromodomain helicase DNA binding protein 5 (CHD5),^63^ a gene activating p53 signaling by targeting p19ARF and impeding p53 ubiquitination degradation.^64^ Knockout of KDM4A from K-Ras-activated A549 cells triggers the senescence of cancer cells.^63^ Interestingly, although KDM4A is present in several subtypes of lung cancer, no correlation between KDM4A content and prognosis has been found in lung cancer, suggesting that KDM4A may be involved only in the early process of lung tumorigenesis.^62^ In addition, KDM4A has been found to regulate other cancer-related genes (*CXCL5*, *ADAM12*, and *JAG1*) by removing methyl groups from H3K9me3 in A549 cells.^62^ The CXCL5 gene has been reported to promote invasion,^65^ metastasis,^66^ proliferation,^67^^,^^68^ radiation resistance,^69^ and angiogenesis in lung cancer.^70^ The *ADAM12* gene is overexpressed in many subtypes of lung cancer and promotes tumorigenesis via proteolytic shedding of EGFR ligands.^71^, ^72^, ^73^, ^74^, ^75^ The *Jagged-1* (*JAG1*) gene has been identified as a diagnostic biomarker for lung cancer and promotes tumorigenesis by mediating the Notch intracellular pathway.^76^^,^^77^ Taken together, these studies suggest that KDM4A promotes lung cancer progression by reducing the tumor suppressor CHD5 and activating related oncogenes.^62^^,^^63^

### KDM4A in breast cancer

KDM4A is overexpressed in ∼60% of patients with breast cancers and forms distinct expression profiles with other KDM4s among different BC subtypes.^78^ For example, KDM4A and KDM4D are co-overexpressed in basal breast cancer, while the KDM4A level is higher in infiltrating breast duct carcinoma (IBDC) than in breast fibroadenoma,^79^ and KDM4B is overexpressed in both estrogen receptor (ER)-positive breast cancer and triple-negative breast cancer.^80^^,^^81^ In IBDC patients, KDM4A levels are negatively associated with tumor suppressor ADP-ribosylarginine hydrolase 1 (ARH1) expression and positively linked with p53 and ER expression.^79^ KDM4A facilitates BC tumorigenesis by activating ERα transcriptional activity although interacting with ERα.^82^ KDM4A inhibition reduces the expression of the ERα target genes *c-Jun* and *cyclin D1* and thus inhibits the proliferation of T47D cells. In HER2 breast leptomeningeal carcinomatosis (HER2 LC), KDM4A promotes cancer growth by increasing *GMCSF* levels.^83^

KDM4A levels are also associated with breast cancer grade, TNM stage, histological type, and disease-free survival (DFS).^84^ Specificity protein 1 (Sp1) is a transcription factor overexpressed in breast cancer, and its expression is negatively associated with BC TNM stage and metastasis status via transcriptionally activating aplasia Ras homologue member I (ARHI). KDM4A reduces the autoregulation of Sp1 and is negatively correlated with tumor suppressor ARHI expression. Consistent with these data, KDM4A inhibition reduces the invasion, migration, and proliferation of MCF-7 cells.^85^ Hypoxia leads to transient site-specific copy gains (TSSGs) independent of hypoxia-inducible factors in a variety of cancer cells.^86^^,^^87^ Hypoxia-driven TSSGs are impeded by succinate-mediated KDM4A inhibition. Several miRNAs (mir-23a-3p, mir-23 b-3p, and mir-137) have also been found to alter KDM4A-mediated TSSG and promote drug resistance in BC cells.^87^ For example, miRNA inhibition promotes TSSGs and up-regulates the drug-resistance protein CKS1B level in primary breast tumors and desensitizes breast cancer cells to cisplatin.^85^

### KDM4A in prostate cancer

Androgen receptor (AR) signaling is a crucial driver of tumorigenesis in prostate cancer (PCa),^88^ a malignant tumor found only in men.^5^ KDM4A is overexpressed in PCa and functions as a coactivator to activate the androgen receptor (AR).^89^ AR is activated by assembling into a complex with KDM4A and thus raises prostate-specific antigen (*PSA*) levels by reducing the binding between H3K9me3 and *PSA* promoters in LNCaP cells.^89^^,^^90^ Additionally, KDM4A is also observed to contribute to PCa cell proliferation through Lgr4/KDM4A/AR signaling by reducing cell apoptosis and inducing cell cycle arrest at the S phase in an androgen-dependent manner.^91^ Moreover, USP1/KDM4A/AR signaling also promotes the proliferation and survival of PCa cells by maintaining the stability of KDM4A.^92^

Apart from AR, KDM4A can also bind to the ETS transcription factor ETV1 and then up-regulate the levels of two downstream effectors by being recruited to the promoters of y*es-associated protein 1* (*YAP1*) and *proteasome 26S subunit non-ATPase 10* (*PSMD10*).^93^^,^^94^ Further study showed that YAP1 and PSMD10 synergistically promote PCa tumorigenesis in a KDM4A demethylase-dependent manner.^94^ Similar to ETV1, the ETS-related gene (ERG) also interacts with KDM4A and promotes PCa progression by increasing YAP1 levels in an H3K9me3 demethylase-dependent manner.^95^ In addition, KDM4A cooperates with ETV2, stimulates the expression of matrix metalloproteinases (MMPs) MMP1 and MMP7, and thus enhances the stemness of LNCaP PCa cells.^96^ Wang et al found that KDM4A can associate with E2F1, enhance the transcriptional activity of the E2F1 target genes pyruvate dehydrogenase kinases (PDKs) *PDK1* and *PDK3* by binding to their promoters, and thus promote cancer cell proliferation and survival by modulating the switch between glycolytic metabolism and mitochondrial oxidation.^97^ Alternatively, two microRNAs are also reported to function as negative regulators (miR-137 and miR-10a) of KDM4A in PCa cells.^98^^,^^99^ miR-137 suppresses PCa tumorigenesis, angiogenesis, and progression by reducing androgen-induced PSA and VEGF expression.^98^ miR-10a functions as a tumor suppressor by negatively modulating the KDM4A-mediated Hippo-YAP1 pathway in PCa.^99^ Moreover, KDM4A assembles into a corepressor complex with nuclear receptor corepressor (NCoR) and HDAC and silences the expression of TRAIL and DR5, which promotes cancer cell survival and desensitizes PCa cells to TRAIL.^61^

### KDM4A in gastric cancer

Gastric cancer (GC) is the leading cancer contributing to global cancer incidence and mortality.^100^ KDM4A is a biomarker for GC diagnosis and prognosis.^101^^,^^102^ Several studies have found that KDM4A not only modulates the growth and invasion of GC by activating the KDM4A/YAP1 pathway^101^^,^^103^ but also sensitizes GC cells to cisplatin, 5-FU, and docetaxel by interacting with pro-apoptotic coiled-coil domain containing 8 (CCDC8).^104^

### KDM4A in hepatoma carcinoma

Hepatocellular carcinoma (HCC) is a kind of malignant tumor with poor clinical outcomes.^105^ KDM4A was found to accelerate the malignant progression of HCC by increasing the levels of *miR-372* and Pim 1 and suppressing p21 expression.^106^ In addition, *miR-24-2* is also reported to promote HCC progression by activating Pim1 by inhibiting KDM4A demethylase activity.^107^ Moreover, *regulatory factor X5*, a key transcription regulator of the *MHCII* gene *in cellulo*, was found to promote HCC progression by transcriptionally up-regulating KDM4A.^108^

### KDM4A in colorectal cancer

Colorectal cancer (CRC) is a heterogeneous disease and one of the leading causes of cancer-related deaths worldwide.^109^ In CRC, KDM4A promotes cell proliferation by interacting with p53 and reducing the transcription of p21, and the further study found that KDM4A sensitizes chemotherapy-induced cell death in both p53-dependent and p53-independent ways.^110^ RAS-association domain family 1 A (RASSF1A) is a kind of RAS effector that regulates cell proliferation and apoptosis,^111^ and KDM4A reduces the transcription of *RASSF1A* by reducing the occurrence of H3K9me2/3 on its promoter and thus promoting cancer progression.^112^ In addition, KDM4A also exhibits tumorigenic activity in a demethylase-independent manner by regulating DNA damage signaling.^113^ Specifically, the Tudor domain of KDM4A competes with the binding site of 53BP1 on H4K20me2 and abrogates 53BP1 recruitment to DNA damage sites, which leads to genomic integrity in CRC cells.

### KDM4A in pancreatic carcinoma

Pancreatic adenocarcinoma (PDAC) is a malignant tumor known as the king of cancer due to its extremely low survival rate.^114^ In PDAC cells, the tumor suppressor miR-137 promotes Ras-induced senescence and activates both the p53 and retinoblastoma pathways by binding and degrading KDM4A mRNA.^115^ In addition, overexpression of the transcription factor RFXAP inhibits the proliferation of PDAC cells by activating the H3K36me3-demethylase activity of KDM4A and thus enhances DNA damage.^114^

### KDM4A in bladder cancer

Bladder cancer is a malignant tumor that causes 170,000 deaths annually worldwide.^116^ KDM4A can assemble into a complex with LSD1 and promote muscle invasion, extravesical extension, and lymph node metastasis of bladder cancer.^117^ Further results showed that KDM4A modulates these processes by transcriptionally activating ADAM12 and SLUG in a demethylase-dependent manner.^62^^,^^118^

### KDM4A in cervical cancer

In cervical cancer, KDM4A was found to promote proliferation and inhibit apoptosis of cervical cancer cells by reducing the tumor suppressor *miR*-*491*-*5p*.^119^ The further study supports that KDM4A up-regulation under hypoxia reduces the occupation of H3K9me3 on the promoters of *hypoxia-induced factor 1α* (*HIF1α*). Then, HIF1α binds to the hypoxia response element (HRE) in the nucleus and activates the transcription and translation of transferrin receptor 1 (TfR1) and divalent metal transporter 1 (DMT1), which induces ferroptosis resistance in cervical cancer cells.^120^

### KDM4A in other cancers

KDM4A is also amplified in other cancers and promotes their progression via a variety of mechanisms. In renal carcinoma, KDM4A or KDM6A raises ribosomal protein-coding genes to sustain tumor cell survival under amino acid deprivation.^121^ Osteosarcoma (OS), a type of bone tumor that seriously affects limb function and leads to great pain in patients, has a poor prognosis due to lung metastasis and chemoresistance. KDM4A mediates drug resistance and lung metastasis of OS by demethylating H3K9me3 at the *SLC7A11* promoter and reducing its transcription, while KDM4A inhibition can induce cell ferroptosis and reverse these processes in OS.^122^ In glioma, KDM4A promotes cell survival by inhibiting autophagy in U87MG and T98G cells.^123^ Mechanistically, KDM4A interacts with DEPTOR and reduces its ubiquitination, and the stability of DEPTOR negatively modulates the mammalian (mechanistic) target of rapamycin (mTOR) 1/2 and inhibits autophagy and apoptosis in glioma.^35^ In head and neck squamous cell carcinoma (HNSCC), KDM4A is overexpressed in HNSCC and lymph node metastasis tissue and promotes the invasion and metastasis of HNSCC.^124^ Mechanistically, transcription factor activating protein 1 (AP-1) facilitates tumor metastasis and growth by occupying the promoters of *JUN* and *FOS* and activating their transcription,^125^ and H3K9me3 inhibits AP-1 activation by occupying the same binding sites. KDM4A rescues H3K9me3-mediated repression via its demethylase activity.^124^ KDM4A is highly expressed in endometrial cancer (EC) and promotes the proliferation, invasion, and metastasis of EC cells by up-regulating *c-MYC* and *AR* and down-regulating *p27*.^126^^,^^127^

## Targeting KDM4A for cancer therapy

Given the crucial roles of KDM4A in tumorigenesis and drug resistance, many pharmacologists and pharmaceutical chemists have been devoted to discovering and developing KDM4A inhibitors to combat KDM4A-mediated cancers. According to the functions and structures of the inhibitors, these KDM4A inhibitors are classified into 7 subcategories: α-ketoglutarate analogues, hydroxamic acids, 8-hydroxy quinoline derivatives, pyridine-based inhibitors, natural products, peptide-based inhibitors, and miscellaneous KDM4A inhibitors.

### α-Ketoglutarate analogues

**Figure 2:**
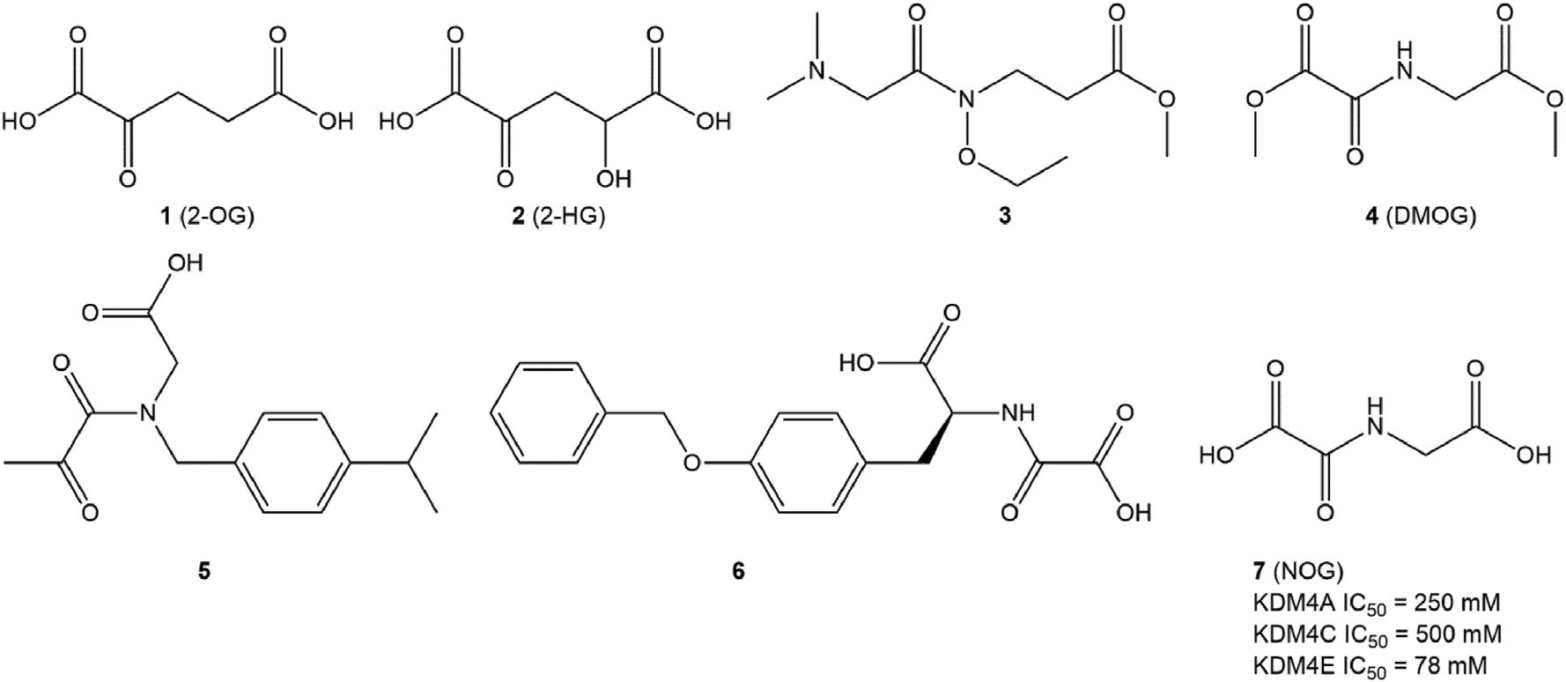
The structure of 2-OG-based KDM4A inhibitors.

α-Ketoglutarate (2-OG; compound 1; Fig. 2) is an indispensable cofactor that maintains the demethylase activity of all JmjC-KDMs, including KDM4A. Therefore, the identification of demethylase inhibitors from 2-OG analogues is a feasible strategy for the development of potent and competitive JmjC-KDM inhibitors. To date, several KDM4A inhibitors (compounds 2–6; Fig. 2) have been identified based on this principle. Among them, NOG (compound 7; Fig. 2), a 2-OG analogue previously identified as a PHD2 inhibitor for HIF, has been found to have weak inhibitory activity against KDM4A by competitively binding to Fe(II) in the catalytic pocket and inhibiting demethylase activity.^128^

### Hydroxamic acids

**Figure 3:**
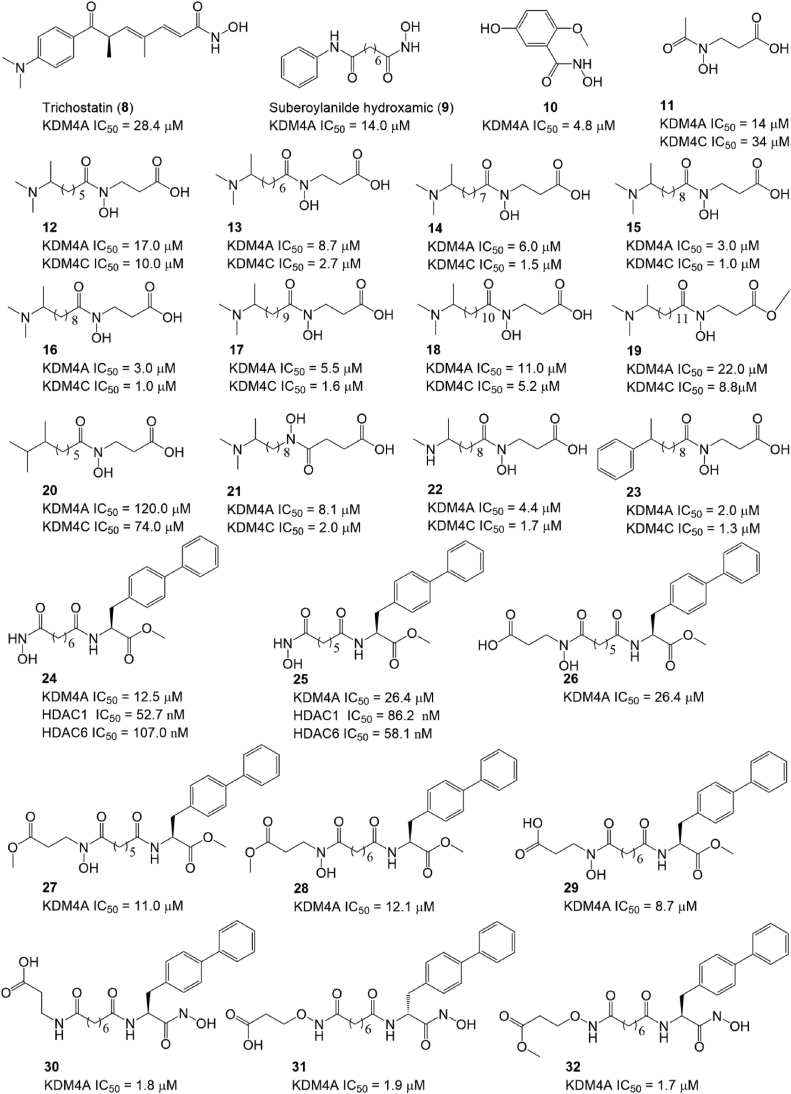
The structure of hydroxamic acids.

Many hydroxamic acids also exhibit good anticancer activity *in vitro* and *in cellulo*. Roles et al reported a set of hydroxamic acids (compounds 8–10; Fig. 3) with IC_50_ values in the range of 4.8–28.4 μM.^129^ Trichostatin (compound 8), first identified as a potent histone deacetylase (HDAC) inhibitor (Ki = 3.4 nM), inhibited KDM4A activity by monitoring demethylation by MALDI-TOF MS. Suberoylanilide hydroxamic (compound 9), another known HDAC inhibitor, was also found to be a KDM4A inhibitor with an IC_50_ of 14.0 μM. Compound 10, the most potent KDM4A inhibitor among them, was verified as a 2-OG completive KDM4A inhibitor. Hamada et al designed a series of hydroxamic acids (compounds 11–23; Fig. 3) initiated with the leading compound 7. Compound 11, a compound produced by replacing the oxalyl moiety of 2-OG with hydroxamate, is a metal ion chelator that is over 10 times more active than compound 7. Then, compounds 12–19 were designed and synthesized by introducing a dimethylamino group and linked with various hydrocarbon chains of different lengths to optimize the potency of compound 11**,** and all 8 compounds showed improved potency against KDM4A with IC_50_ values ranging from 3.0 to 22.0 μM. Compound 15 was a compound with a 250-fold and 500-fold improvement in inhibitory activity against KDM4A and KDM4C relative to compound 7, respectively.^130^ Compound 16, a methylated derivative of compound 15, has similar *in vitro* inhibitory activities against KDM4A and KDM4C but improved anti-proliferative and anti-transformation activities against breast cancer cells.^81^ To explore the importance of the amino group of these compounds, compound 20 was designed with the terminal nitrogen of compound 12 replaced by a carbon, and its KDM4A-inhibitory activity is much less potent than compound 12. Compound 21 was synthesized to further investigate the structure–activity activity by converting the hydroxamate of compound 15 to the retro-hydroxamate, and this alternation decreased KDM4A-inhibitory activity by 2-fold compared with compound 15. Then, the importance of the dimethylamino group was also explored by replacing this group with other alkylamino groups (compounds 22, 23; Fig. 3). The results showed that these conversions only slightly affected KDM4A-inhibitory activity compared with compound 15.

Morera et al found that a previously reported HDAC inhibitor compound 24 also had weak KDM4A-inhibitory activity with an IC_50_ of 25.4 μM. They designed a series of compounds (compounds 25–32; Fig. 3) to improve potency and selectivity and explored their SAR.^131^ Compound 32 is the most potent KDM4A inhibitor, with an IC_50_ of 1.7 μM, as detected by the LANCEUltra assay. Compared with the IC_50_ values of compounds 24 & 25, 27 & 28, and 26 & 29, the linker hydrocarbon chains were found to have only a slight effect on KDM4A inhibition. Then, the importance of cap hydroxamic acids was investigated by replacing the hydrogen with acetate or the methyl acetate group, and the results indicated that the cap modifications also had a slight effect on improving the potency and selectivity of the series (compounds 24–29). Further SAR analysis found that the replacement of the ether methyl group improved KDM4A-inhibitory activity by 4–7-fold (compounds 27–32; Fig. 3). Further studies also verified that compounds 27–32 also exhibited anti-proliferative activity in cancer cells.

### 8-Hydroxy quinoline derivatives

**Figure 4:**
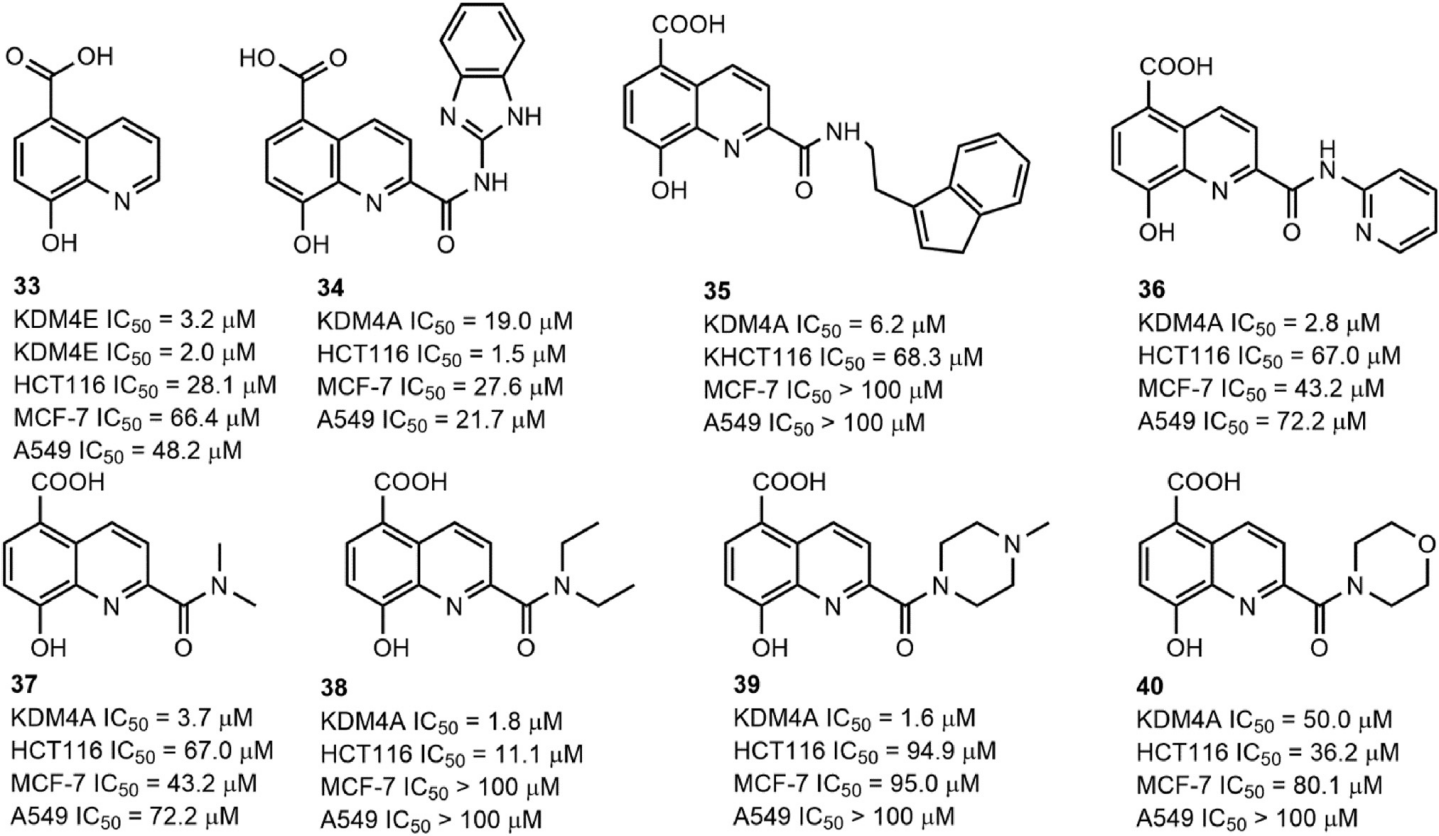
The structure of 8-hydroxy quinoline derivatives as KDM4A inhibitors.

5-Carboxy-8-hydroxyquinoline (compound 33; Fig. 4) was reported to suppress KDM4A demethylase activity by chelating with Ni(II) in a bidentate fashion via its 8-hydroxy group and quinoline nitrogen with an *in vitro* IC_50_ value of 3.2 μM. However, compound 33 exhibited low cytotoxicity to cancer cells, probably due to its poor cell permeability.^132^ To improve the *in cellulo* anticancer activity, Feng et al designed a set of derivatives (compounds 34–40; Fig. 4) of compound 33 by introducing an appropriate substitution at C-2. All 9 compounds showed *in vitro* IC_50_ values in the micromolar range (1.6–50.0 μM) against KDM4A. Interestingly, although compounds 35–39 exhibited improved *in vitro* KDM4A-inhibitory activity against compound 34, compound 34 had the best anti-proliferative activity against different cancer cells. The further study verified that good aqueous solubility and appropriate cell permeability contributed to this phenomenon.

### Pyridine-based inhibitors

**Figure 5:**
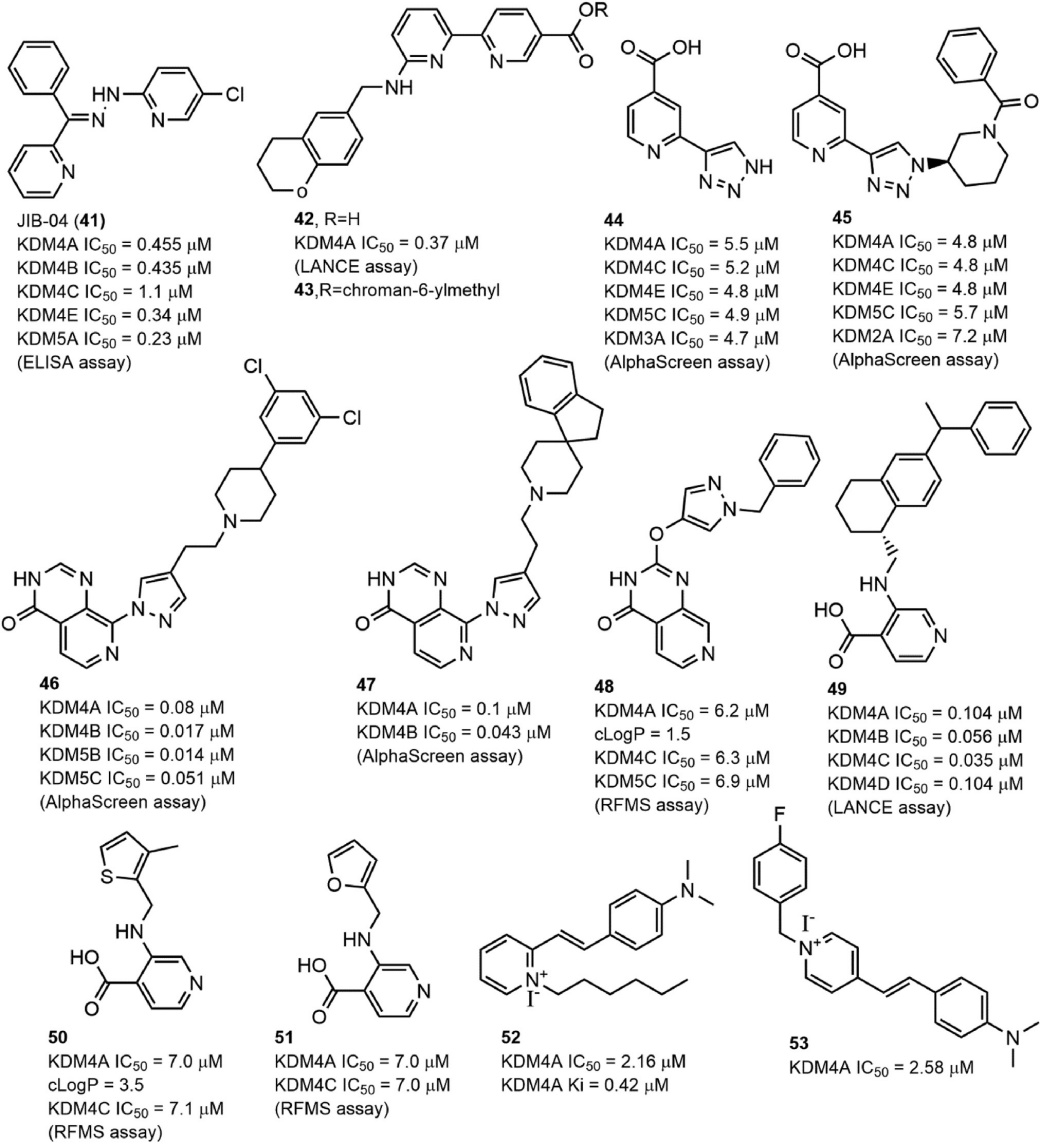
The structure of pyridine-based KDM4A inhibitors.

Pyridine-based inhibitors exhibit good *in vitro* potency against KDMs by chelating metal ions within these histone demethylases by their pyridine moieties. Therefore, the pyridine group has also been widely used to design KDM inhibitors, including KDM4A inhibitors.^3^^,^^4^^,^^133^^,^^134^ JIB-04 (compound 41; Fig. 5) was first identified as a pan-JmjC KDM inhibitor using a cell-based assay.^135^ It showed micromolar inhibitory activity against KDM4s and KDM5A. Further study indicated that compound 41 antagonized KDM4A in a Fe^2+^-binding mode, validated by which the Z-isomer of compound 41 without a pyridine moiety lacked inhibitory activity. Compound 41 also exhibited potent anticancer activity and suppressed tumor growth in a tumor-burdened mouse model by modulating KDM4A activity *in vivo*. Compound 42 was identified as a potent KDM4A inhibitor with an IC_50_ of 0.94 μM using *in silico* screening and structure-based optimization.^136^ It also exhibited good inhibitory activity against KDM5A (IC_50_ = 0.44 μM) and KDM6B (IC_50_ = 36.5 μM). The co–crystal structure showed that the nitrogen atoms of pyrimidine and pyridine chelated the Fe^2+^ within KDM4A, while the carboxylate group interacted with K206 and Y132, and the secondary amine formed a hydrogen bond with the carboxylate of E190. The terminal pyridine ring inserts into a large binding pocket formed by V171, Y175, and D191. Compound 43 is an ester derivative of compound 42 produced by introducing a chroman-6-ylmethyl group in its side chain, and it exhibited improved solubility and anti-proliferative activity in KYSE-150 cells. Compound 44, a 2,4-PDCA derivative produced by replacing the C2 carboxylate group with a triazole group, showed inhibitory activity towards KDM4s, KDM5A, and KDM2A (IC_50_ = 4.8–5.7 μM). The co–crystal structure between KDM4A and compound 44 revealed that the nitrogen-containing heterocyclic within compound 44 exhibited a similar role as pyridine in 2,4-PDCA.^137^ In addition, modification of the moieties of the heterocyclic ring (compound 45; Fig. 5) also slightly improved the selectivity and potency of compound 44 against KDM4s. Compounds 46 and 47 were two 2-OG–substrate mimics designed starting from a 2-OG mimic.^138^ The docking analysis indicated that the chlorobenzyl piperidine group of compound 46 protrudes into the substrate-methylated lysine side sub-pocket, which may contribute to its inhibitory activity towards KDM4s (IC_50_ values 17 nM–80 nM). Moreover, the introduction of a pyrido [3,4-d]pyrimidin-4(3H)-one group significantly improved its cellular permeability. Compound 47 had KDM4A inhibitory activity similar to that of compound 46. A change in the position of the side chain (compound 48) significantly reduced the inhibitory activity against KDM4A.^139^ Westaway et al developed 3-amino-4-pyridine carboxylate derivatives (compounds 49–51; Fig. 5) with KDM4A inhibitory activity.^140^ Among them, compound 49 had the best inhibitory activity against KDM4A *in vitro*, and all of them had good cell penetration, as indicated by an inhibitory activity of less than 100 nM *in cellulo*. Further study showed that they chelated Fe^2+^ by their nitrogen of pyridine rings, and the carboxylate group at the C4 position and the amino group linked to form a bicyclic scaffold contributed to maintaining their potency. Wang et al also identified 2 pyridine-based KDM4A inhibitors, compounds 52 and 53**,** with moderate inhibitory activity (IC_50_ = 2.58 μM and 2.16 μM, respectively) using an unbiased high-throughput screening approach from a chemical library containing 14,400 compounds based on a fluorescence polarization (FP)-based competitive binding assay.^141^ Further study showed that compounds 52 and 53 not only inhibited KDM4A in a non-chelated manner *in vitro* but also induced the accumulation of hypermethylated substrates in cells. Considering that the two compounds shared a pyridinium fragment and a dimethylamino-styrene fragment, this scaffold provides a foundation for further optimizing and designing more potent and selective KDM4A inhibitors.

### Natural products

**Figure 6:**
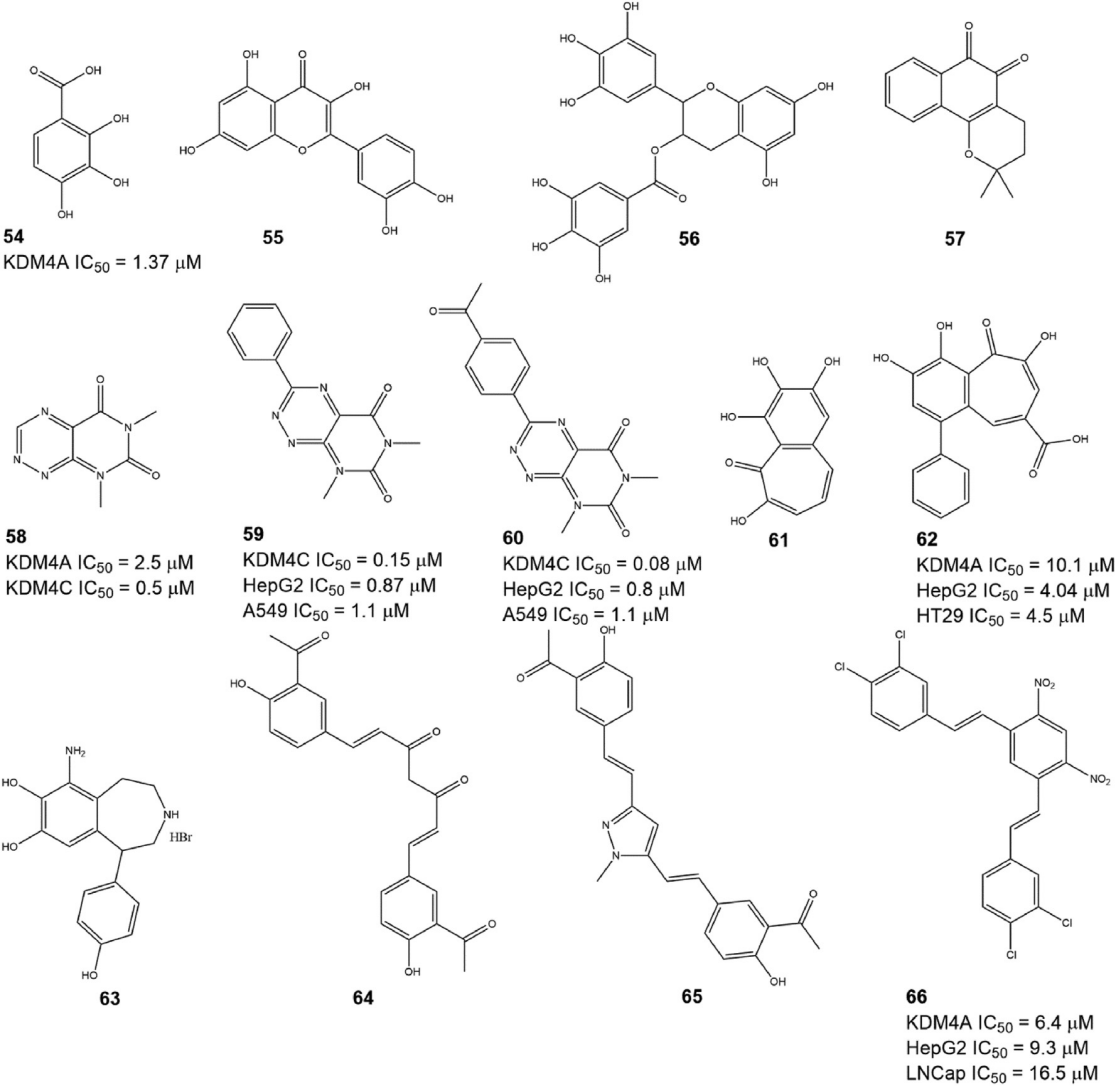
The structure of natural products as KDM4A inhibitors.

Natural products are one of the major sources for discovering epigenetic modulators due to their diversity in structure and activity.^142^, ^143^, ^144^ To date, over 10 natural products have been found to have KDM4A-inhibitory activity. Feng et al identified several KDM4A inhibitors (compounds 54–57; Fig. 6) based on a hierarchical workflow combining shape- and electrostatic-based virtual screening from a natural product library and docking analysis indicated that compound 54 (IC_50_ = 1.37 μM) has a similar binding mode as other 2-OG completive inhibitors.^145^ Plant flavones 55 and 56 containing a catechol scaffold also exhibited inhibitory activity against KDM4s by increasing H3K9me3 levels *in cellulo*.^146^^,^^147^ Toxoflavin (compound 58, also named PKF118-310) was first identified as a transcription factor 4 (TCF4)/β-catenin signaling inhibitor, and this compound and its derivatives (compounds 59 and 60) also have KDM4A inhibitory activity based on a structure-based virtual screening.^148^ Further study showed that compound 58 could also suppress the proliferation of HCT-116 and U937 cells by inducing cell cycle arrest and apoptosis through increasing H3K9me3 levels. Unfortunately, the high redox liability of compound 58 induced peroxide formation, which limited its further advancement into the clinic. To solve this drawback of compound 58, a set of C3 derivatives of compound 58 were designed and synthesized, and two of them (compounds 59 and 60) showed improved KDM4 inhibitory activity, cell permeability, and metabolic stability.^149^ Purpurogallin (compound 61), a natural product with a benzotropolone scaffold and potential anticancer activity, was found to have inhibitory activity against KDM4s.^150^ Further optimization showed that the introduction of a carboxylic acid moiety at the C4 position of the benzotropolone scaffold (compounds 62 and 63) and a halogen group in the benzene nucleus could increase the activity of compound 61, but the carboxylic acid in these optimized compounds reduced their cell permeability, while the replacement of the halogen with a phenol group could increase the cell permeability of compound 61 and inhibit the proliferation of several cancer cells. Curcuminoids (compound 64–66) were found to selectively inhibit some isoforms of KDM4s.^151^ Compound 66 is a KDM4A inhibitor derived from the lead compound curcumin (compound 64), and it induced the cell death of Hep G2 and LNCaP cells in a KDM4A/B demethylase-dependent manner.^152^

### Peptide-based inhibitors

**Figure 7:**
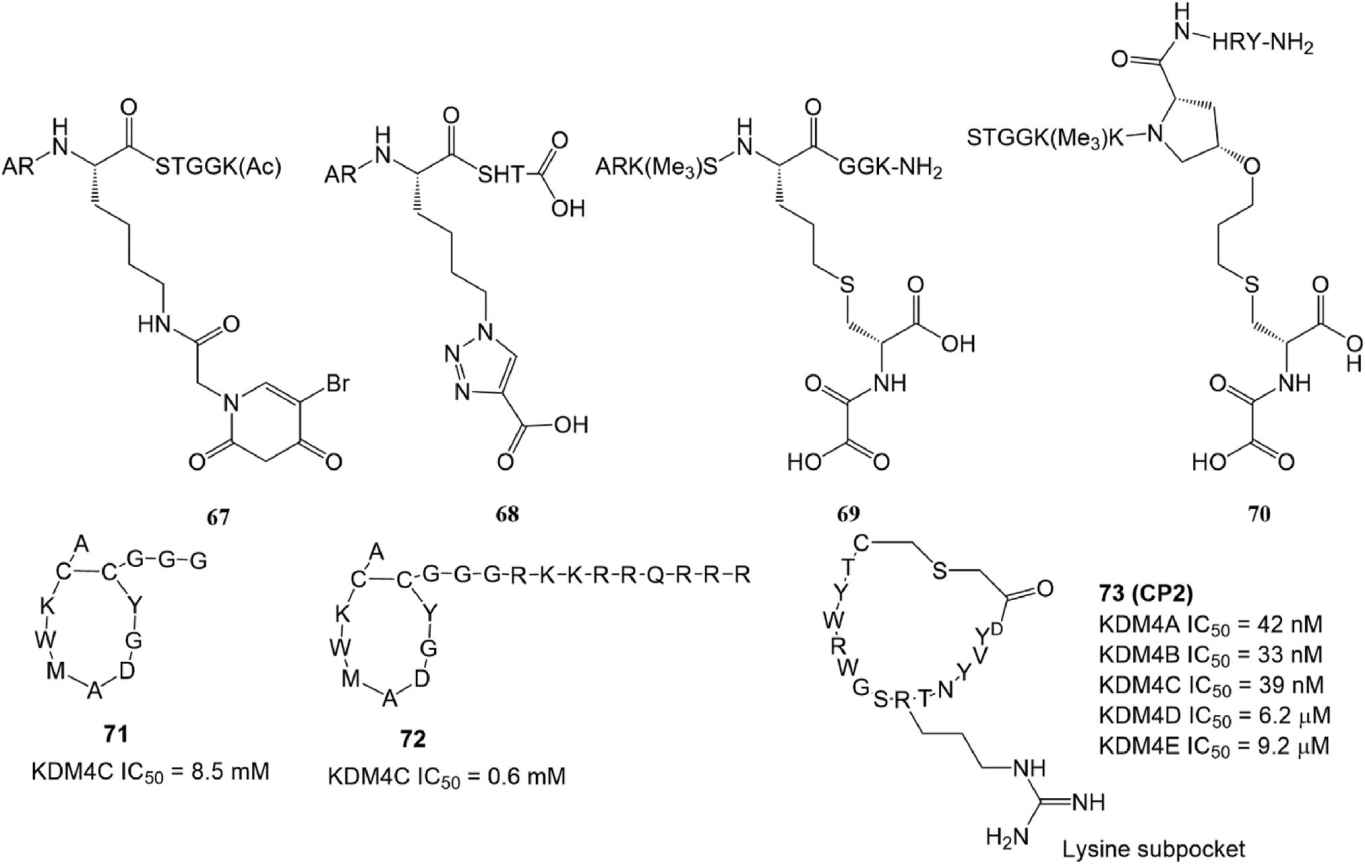
The structure of peptide-based KDM4A inhibitors.

Peptides are often used to develop epigenetic enzyme inhibitors by truncating or modifying their substrate proteins.^153^ A truncated H3K9me3 designed by Lohse et al even exhibited much better KDM4A affinity (*K*_cat_ = 0.01 min^−1^, *K*_M_ = 121 μM) than endogenous substrate^154^ A previous study suggested that modifying H3K9me3 mimic peptides by introducing different functional groups into their Lys 9 side chain would improve their selectivity for KDM4A. Two of these compounds, 67 (adding a metal-chelated group) and 68 (introducing a carboxylate group), exhibited better KDM4A inhibitory activity *in vitro* and *in cellulo*.^154^ Woon et al found that introducing a NOG group at position 10 of the H3K9me3 peptide (compound 69) or Pro 38 of the H3K36me3 peptide (compound 70; Fig. 7) could also increase the potency and selectivity of KDM4A and KDM4C.^155^ Cyclic peptides are favored by pharmaceutical chemists due to their great binding affinity, low toxicity, and capability of targeting traditionally “undruggable” protein surfaces.^156^ Several cyclic peptide-based KDM4 inhibitors (compounds 71–73; Fig. 7) were developed based on diverse designs and screening strategies.^157^^,^^158^ Among them, compounds 71 and 72 inhibited KDM4C in a substrate- and 2-OG-independent manner, which provided novel strategies to discover selective KDM4C inhibitors.^157^ Compound 73 (CP2)**,** another macrocyclic peptide, selectively inhibited KDM4A-C with IC_50_ values of 42 nM, 33 nM, and 39 nM, respectively. In addition, compound 73 exhibited exceptional selectivity within the KDM4 subfamily with over 100-fold greater potency against KDM4A-C than against KDM4D-E.

### Miscellaneous KDM4A inhibitors

**Figure 8:**
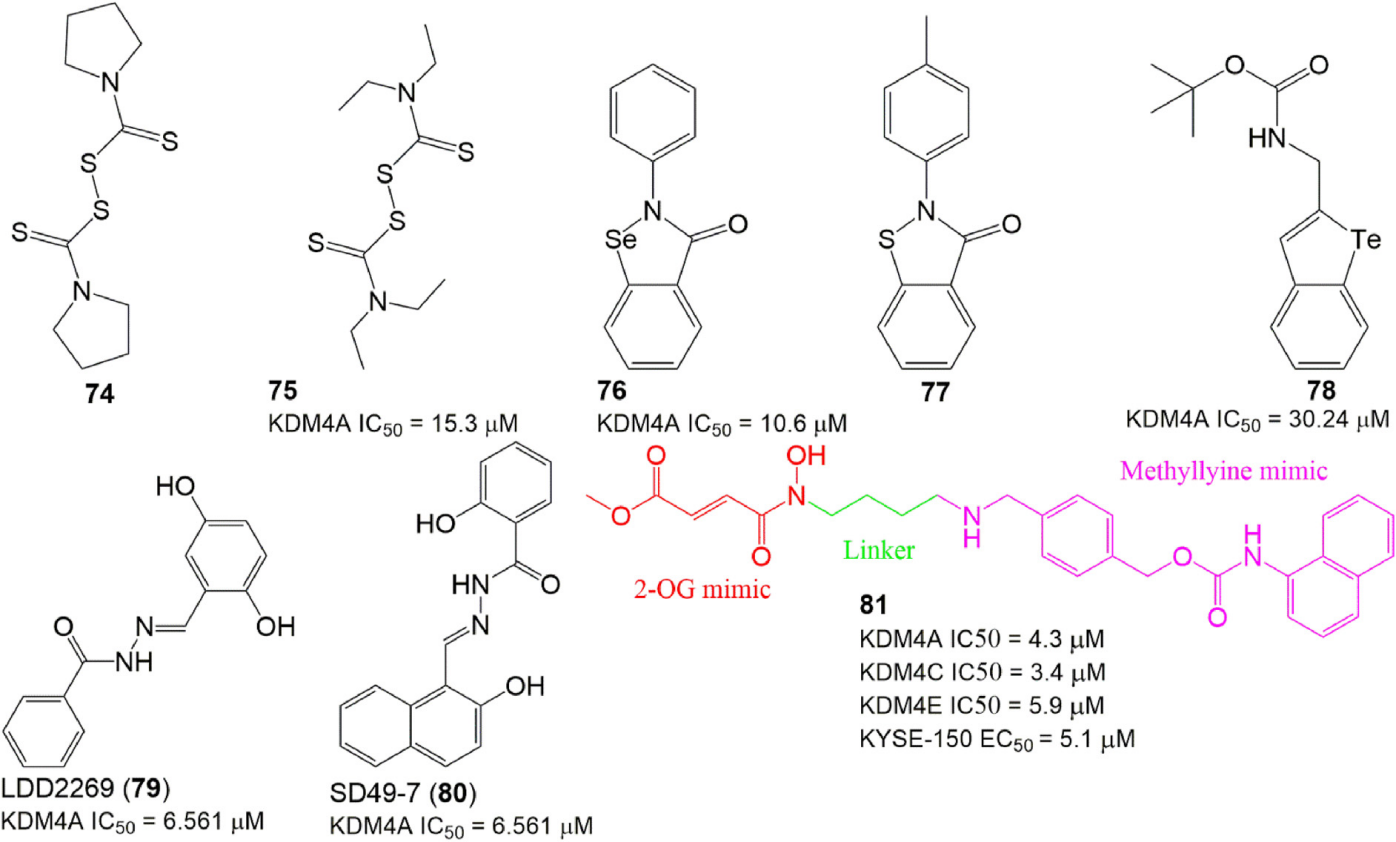
The structure of miscellaneous KDM4A inhibitors.

The zinc-binding site is a unique structural motif (His 220, Cys 234, Cys 306, and Cys 308 in KDM4A) in the vicinity of the active site of KDM4s and is pivotal for peptide recognition but without any apparent interactions.^159^ Four organoselenium/sulfur compounds (compounds 74–77; Fig. 8) were identified as KDM4A inhibitors by ejecting zinc ions.^160^ Among them, compounds 75 (disulfiram) and 76 (ebselen) exhibited moderate KDM4A inhibitory activity with IC_50_ values of 3.3 μM and 10.6 μM, respectively. However, because zinc ions are essential for many zinc-dependent metal proteins, the selectivity of these kinds of KDM4A inhibitors has yet to be investigated. Kim et al identified a t-butyl carbamate derivative 78 as a KDM4A inhibitor with an IC_50_ of 30.24 μM and cytotoxicity to HeLa cells. Interestingly, the corresponding selenium or oxygen-substituted compounds of 78 did not display any inhibitory activity towards KDM4A.^161^ LDD2269 (compound 79) is a KDM4A inhibitor with an IC_50_ of 6.5 μM.^162^ Molecular docking analysis showed that it could form two hydrogen bonds with KDM4A (residues Asp 135 and Asp 191) via its 2,5-hydroxyl group of the 2,5-dihydroxybenzyl moiety, a strong π–cation interaction with Lys 241 of KDM4A via its benzyl group of the 2,5-dihydroxybenzyl moiety, and π–π interactions with Phe 185 via the benzyl group of the R2 position. Further study showed that compound 79 could exhibit its anticancer activity by inhibiting cell proliferation through induced cell apoptosis in HCT116 cells. Recently, Li et al found that SD49-7 (compound 80) was a KDM4 inhibitor with good *in vitro* and *in vivo* anti-leukemic activity.^163^ Mechanistically, compound 80 administration could activate apoptosis signaling by reducing *MDM2* levels by regulating the occupation of H3K9me3 levels on the *MDM2* promoter region. Hybrid molecule 81 is a KDM4A inhibitor designed using a “two-component” strategy by crosslinking a 2-OG mimic KDM4A inhibitor with a methyl lysine mimic.^164^ Although compound 81 exhibited much better inhibitory activity against KDM4A than its two parent compounds, it suffered from poor selectivity for the KDM4 subfamily.

## Discussion and prospects

As an epigenetic transcriptional regulator, an increasing number of studies have shown that KDM4A plays a dual role in tumorigenesis by mediating downstream gene activation or repression.^7^^,^^9^ Although there are many reports on the roles of KDM4A in cancer progression and development, more studies are still necessary to explore the precise functions of KDM4A in a variety of cancers due to their heterogeneity. Moreover, most previous studies have mainly focused on the interaction between KDM4A and its client proteins on the demethylase activity of KDM4A, and there are few reports about the effects of epigenetic modifications within critical amino acid residues of KDM4A on its demethylase activity and its mediation of diseases. The epigenetic modifications of KDM4A are also associated with tumor progression. For example, FBXO22 functions as a receptor for KDM4A by recognizing its catalytic JmjN/JmjC domains via its intracellular signal transduction (FIST) domain and thus induces the ubiquitin-mediated proteasomal degradation of KDM4A.^30^ Moreover, there are also limited advances in understanding the non-catalytic activity of KDM4A compared to its demethylase activity. For example, although PHD domains in other KDMs are able to bind modified and unmodified histone residues, whether the two PHDs have similar activity is still unclear.^165^ Therefore, it is imperative to investigate the underlying mechanisms of these non-catalytic domains in mediating tumorigenesis.

Although tens of KDM4A inhibitors have been reported to date, none of them have advanced into the clinic. There are several obstacles to overcome before KDM4A inhibitors can see clinical use. First, like other JmjC demethylase inhibitors, the current KDM4A inhibitors mainly target catalytic domain binding inhibitors. Unfortunately, the JmjC domain is shared by all JmjC demethylases and is highly conserved among different demethylases, which inevitably leads to off-targeting effects and increases the difficulty in developing highly selective KDM4A inhibitors.^4^ Allosteric regulation is a common characteristic for various enzymes,^5^^,^^48^^,^^166^, ^167^, ^168^, ^169^, ^170^ and it is thus feasible to develop new allosteric agents to improve the selectivity of KDM4A inhibitors. In addition, targeting protein–protein interactions (PPIs) is an effective strategy to improve selectivity and reduce off-target risks.^48^^,^^143^^,^^171^, ^172^, ^173^, ^174^ Due to KDM4A mediating tumorigenesis by interacting with distinct ligand proteins, blocking the PPI between KDM4A and its client proteins is also an alternative to improve the selectivity of KDM4A inhibitors. Second, because of the heterogeneity of KDM4A-mediated cancers, it is imperative to identify the anticancer profile of KDM4A inhibitors to progress them into clinical use for precision treatment. Third, although over 70 KDM4A inhibitors have been found, most of them have low permeability and stability *in cellulo* and *in vivo*, which greatly limits further applications. Computer-aided virtual screening and nanocarrier-mediated drug delivery are two methods that improve cell penetration and drug stability,^173^^,^^175^ and they are potential strategies to optimize the permeability and stability of KDM4A inhibitors. Fourth, the currently limited scaffolds also reduce the opportunity to discover more selective and potent KDM4A inhibitors. Natural products are a unique source of active molecules for epigenetic drug discovery,^142^^,^^171^^,^^176^ and some plant flavones also exhibit inhibitory activities against KDM4A,^145^, ^146^, ^147^ suggesting the possibility of discovering more selective and potent KDM4A inhibitors from natural products. In addition, our previous studies found that rhodium/iridium complexes are important sources for the discovery of various enzyme inhibitors with excellent biocompatibility and *in vitro* potency,^174^^,^^177^^,^^178^ and an identified rhodium-based KDM5A inhibitor by our group also showed weak KDM4A inhibitory activity at 3 μM,^177^ indicating that metal complexes are also potential sources for designing KDM4A inhibitors. Fifth, KDM4A is also found to mediate drug resistance to several clinical chemical agents, and abrogating its demethylase activity will restore or sensitize cells to the anticancer activity of chemical drugs, suggesting that single administration or combined therapy using KDM4A inhibitors and chemical agents is a feasible strategy to treat drug-resistant cancers.

In summary, KDM4A inhibition is an effective strategy to treat multiple KDM4A-overexpressing cancers and combat many KDM4A-induced drug-resistant cancers. Further exploration of the biological functions of KDM4A in cancer and adaptation profiles of KDM4A inhibitors will advance the applications of KDM4A inhibitors for clinical use in the future.

## Author contributions

G. Yang collected and collated documents and wrote the manuscript. C. Li, F. Tao, Y. Liu, M. Zhu, and Y. Du collected and collated documents and wrote the manuscript. C. Fei revised and modified the manuscript. Q. She and J. Chen collected and collated documents and wrote and proofed this review. All authors read and approved the final version of the manuscript.

## Conflict of interests

The authors declare no conflict of interests.

## Funding

This work is supported by the National Natural Science Foundation of China (No. 31972821), the General Scientific Research Project of Education of Zhejiang Province, China (No. 422204123), and the Starting Research Fund of Ningbo University, Zhejiang, China (No. 421912073).
